# Development of Personalized Strategies for Precisely Battling Malignant Melanoma

**DOI:** 10.3390/ijms25095023

**Published:** 2024-05-04

**Authors:** Armond J. Isaak, GeGe R. Clements, Rand Gabriel M. Buenaventura, Glenn Merlino, Yanlin Yu

**Affiliations:** Laboratory of Cancer Biology and Genetics, Center for Cancer Research, National Cancer Institute, National Institutes of Health, Bethesda, MD 20892, USA

**Keywords:** melanoma, metastatic treatment, precision oncology, targeted therapy, vaccine, immunotherapies, adoptive cell therapy, personalized medicine

## Abstract

**Simple Summary:**

Precision oncology is emerging as a viable option to improve survival in patients who fail to remain disease-free even after the employment of currently available treatments. Such an alternative is particularly essential for patients with melanoma, where recurrence and progression of the disease following the development of acquired resistance are common. In this review, we aim to address the treatments available to patients diagnosed with melanoma and discuss promising treatment options that rely on specific and individualized approaches to treatment, particularly for the subset of patients who experience therapy failure due to acquired resistance.

**Abstract:**

Melanoma is the most severe and fatal form of skin cancer, resulting from multiple gene mutations with high intra-tumor and inter-tumor molecular heterogeneity. Treatment options for patients whose disease has progressed beyond the ability for surgical resection rely on currently accepted standard therapies, notably immune checkpoint inhibitors and targeted therapies. Acquired resistance to these therapies and treatment-associated toxicity necessitate exploring novel strategies, especially those that can be personalized for specific patients and/or populations. Here, we review the current landscape and progress of standard therapies and explore what personalized oncology techniques may entail in the scope of melanoma. Our purpose is to provide an up-to-date summary of the tools at our disposal that work to circumvent the common barriers faced when battling melanoma.

## 1. Introduction

**Figure 1:**
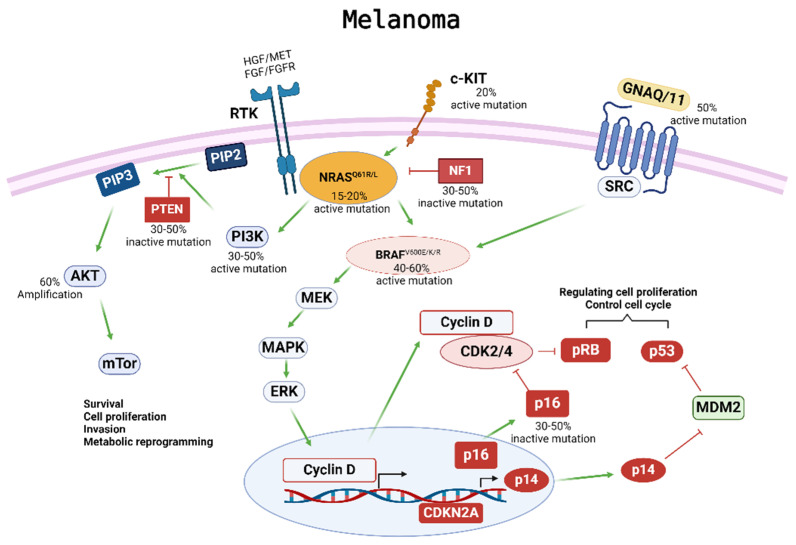
Melanoma development and progression are driven by multiple driver gene mutations. Four major subtypes of cutaneous melanoma have been established according to specific somatic mutations in different oncogenes, including BRAF, NRAS, NF1, c-KIT4, and GNAQ/11. These somatic mutations activate the phosphoinositide 3-kinase/protein kinase B (PI3K/AKT) and mitogen-activated protein kinase (MAPK) pathways, resulting in melanoma development and progression. Mutations in BRAF can impact the MAPK pathway and have downstream effects on cell proliferation and cell cycle control. Mutations in NRAS can affect both the MAPK and the PI3K/AKT pathways, which regulate cell survival, proliferation, invasion, and metabolic programming. Overactivation of these pathways can contribute to the tumorigenesis, proliferation, invasion, and metastasis of melanoma cells, as well as drug resistance to applied therapies.

Cutaneous melanoma remains the most aggressive and deadliest form of skin cancer, with a survival rate of less than 10%, despite recent advances in therapy [1]. Four major subtypes of cutaneous melanoma have been established according to the presence of specific somatic mutations occurring in different oncogenes (Figure 1): (1) the B-Raf proto-oncogene serine-threonine kinase (BRAF) mutant, (2) the NRAS proto-oncogene GTPase (NRAS) mutant, (3) the neurofibromin-1 (NF1) tumor suppressor mutant, and (4) the triple BRAF/NRAS/NF1 wildtype (WT) form that does not contain mutations in any of the three oncogenes [2,3]. An overwhelming proportion of melanomas harbor hotspot mutations in either the BRAF or NRAS oncogenes—over 50% and approximately 20–25% of melanomas, respectively [4,5,6]. Patients with metastatic BRAF-mutant melanoma have an estimated survival of 6–10 months after diagnosis without current drug therapy intervention [7].

Each melanoma case is categorized based on the 2009 TNM (Tumor, Node, Metastasis) staging system derived by the American Joint Committee on Cancer (AJCC). The AJCC system stages disease on a scale of stage I–V, where patients may present with solely a primary tumor/local disease (I–II), node-positive disease (III), or advanced or metastatic disease (IV). These stages consider the extent of the primary tumor in terms of thickness and the possible presence of ulceration (T), an investigation of spread to nearby lymph nodes (N), and metastatic spread (M) [1,8]. Although surgical resection can successfully treat patients with melanoma in the earlier stages [3], those diagnosed with stage IV/late-stage, metastatic, or unresectable melanoma have historically faced limited treatment options and dismal prognoses. An optimal treatment strategy is based on somatic mutational status and the patient’s clinical presentation (e.g., toxicity profiles, disease dissemination, serum lactate dehydrogenase) [4,9]. To date, two types of systemic therapies have been identified as the most efficacious treatment option for advanced and high-risk early-stage melanoma: targeted therapy and immune checkpoint inhibitors (ICIs). Targeted therapy employs small-molecule inhibitors, notably BRAF kinase and mitogen-activated protein kinase inhibitors (BRAFis and MAPKis), selectively designed to inhibit targets in the MAPK pathway [10]. Meanwhile, the development of ICIs allows for the pharmacological manipulation of the immune response, reactivating T cells to inhibit tumor cell evasion [9].

Cutaneous melanomas have high mutational burdens, making this disease a prime candidate for adoptive cellular therapy (ACT) of either tumor-infiltrating lymphocytes (TILs) or chimeric antigen receptor T (CAR-T) cells [1,11,12,13,14]. From a treatment perspective, TIL therapy is behind MAPKi targeted therapy and ICI therapy, especially for treatment-refractory patients. CAR-T therapy may be particularly helpful for metastatic and targeted-therapy-resistant melanoma patients [1,11,12]. However, while it has higher specificity than MAPKi targeted therapy, CAR-T therapy has more dangerous potential side effects, such as cytokine release syndrome (CRS) [11,12,14]. The review aims to recapitulate an up-to-date overview of where we stand regarding the scope of precision oncology techniques, describe what we can expect in terms of the future landscape by challenging what is already known in the field, and provide recommendations for ways to circumvent the crucial dead ends we currently face in the treatment of advanced melanoma.

## 2. Biomarkers for Personalized Targeted Therapy

The molecular composition of the tumor and the tumor microenvironment (TME) play an important role in the progression of cutaneous melanoma. The different subtypes of melanoma can vary widely in their properties, consequently affecting treatment and patient survival benefits.

### 2.1. BRAF and MEK-Related Biomarkers

BRAF, along with CRAF, is a member of the Raf family protein and plays a role in the MAPK signaling pathway via downstream activation of MEK and, subsequently, ERK. BRAF is a serine-threonine-specific kinase whose most common mutation occurs at codon 600 in melanoma. Approximately 80% of these missense mutations involve the substitution of a Valine (V) for a Glutamate (E) in the activation loop, known as BRAF^V600E^ mutations [4]. The remaining portion of mutations can include V600 substitutions for a Lysine (K) (BRAF^V600K^) or an Arginine (R) (BRAF^V600R^) [4]. Importantly, these substitutions initiate a constitutional activation of the MAPK pathway, leading to the overactive growth of BRAF-mutant melanoma cells. BRAF mutations typically develop from external environmental causes (e.g., UV radiation from the sun), and as such, it is very rare for this to be an inherited mutation [15].

Patients with BRAF-mutant melanomas have historically demonstrated an initial benefit from the pharmacological inhibition of mutant BRAF and/or MEK. Current targeted therapies include the first FDA-approved BRAF inhibitors (BRAFis) vemurafenib (PLX-4032, approved in 2011) and dabrafenib (GSK2118436, approved in 2013) [16]. These BRAFis can be taken orally and have high selective binding to the ATP-binding site of BRAF mutants V600E, V600D, V600K, and V600R [15]. Multiple studies have shown that both vemurafenib and dabrafenib improved patient outcomes when used as monotherapies against BRAF^V600E^ unresectable or metastatic melanoma relative to the prior therapy standard of chemotherapy [17,18]. The most recent BRAFi to gain FDA approval was encorafenib in 2018 [19]. In tandem with BRAFis, MEK inhibitors (MEKis) have been developed to target a downstream molecule of the BRAF kinase and produce a longer-lasting effect on progression-free survival (PFS). There are currently three main FDA-approved MEKis: trametinib (2013), cobimetinib (2015), and binimetinib (2018) [16,20]. MEKis can target both forms of MEK (MEK1 and MEK2), generally through allosteric binding that inhibits MEK recruitment, phosphorylation, release, or affinity with BRAF [21]. As with BRAFi monotherapy, pharmacological inhibition by the MEKi trametinib has been shown to have a significant survival benefit compared to chemotherapy [22]. Treatment with BRAFis and MEKis is not without difficulty, as patients typically fail to remain disease-free long term due to the development of resistance to these inhibitors. Using BRAFis and MEKis in combination has yielded some improvements—common combinations include dabrafenib and trametinib, vemurafenib and cobimetinib, and encorafenib and binimetinib [23]. However, drug resistance is still prevalent and has become a critical factor in the search to develop new immunotherapies. 

**Figure 2:**
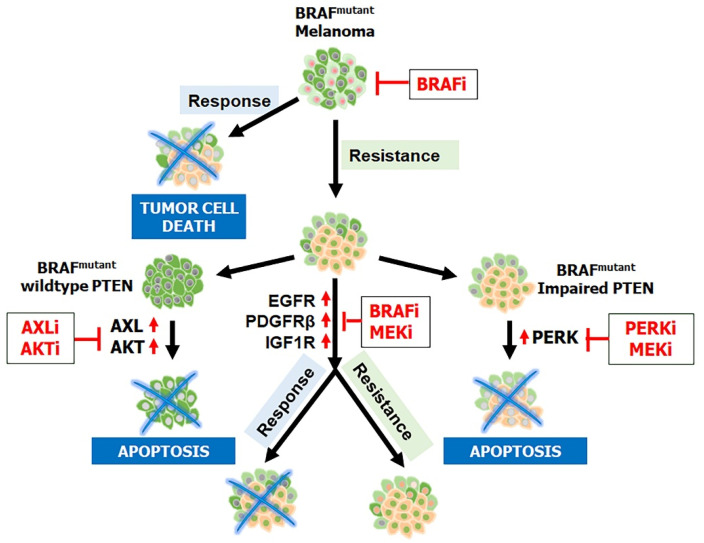
Targeted therapy and mechanism of resistance. Treatment with BRAFis causes programmed cell death in melanoma patients whose cancer has a BRAF^V600E^ mutation. Resistance to BRAFis can arise as tumor cells overexpress EGFR, PDGFRβ, and IGF1R, creating an alternative survival pathway. Treatment with a combination of BRAFis–MEKis can result in programmed cell death but may also lead to double resistance to these therapies. The molecular mechanism mediating resistance may depend on PTEN status in BRAF-mutant melanoma. In melanoma with wild-type PTEN, AXL and AKT activation are elevated and can confer resistance to BRAFis; treatment with AXLi and AKTi can block further tumor growth. In contrast, melanoma with impaired PTEN is associated with decreased AKT activation; the MAPK/ERK pathway is reactivated as the core drug resistance pathway and is responsive to treatment with PERKis and MEKis.

Furthermore, clinicians have found that having a BRAF mutation alone is insufficient to cause melanomagenesis; an inactivating PTEN (phosphatase and tensin homolog deleted from chromosome 10) mutation is also required [24,25]. PTEN, a protein that works in opposition to PI3K to dephosphorylate PIP3 to PIP2, is also known to inhibit BRAF’s kinase expression and therefore regulate downstream MAPK signaling [25,26]. Evidence indicates an association between BRAF and PTEN mutations, with PTEN inactivation being one of the most common causes of resistance to BRAF inhibitors [27,28,29,30] (Figure 2). Catalanotti et al. [31] observed similar findings where patients with PTEN loss-of-function melanoma had reduced survival and response rates. Moreover, melanomas with inactivation of PTEN or activated PI3K/AKT have a higher potential of metastasis [32,33,34]. These results highlight the potential of PI3K inhibitors, in combination with BRAF and MEK inhibitors, to treat BRAF-mutant patients regardless of their PTEN status.

To circumvent BRAFi and MEKi double resistance, their upstream and downstream targets are also a focus for clinicians. For example, SHP2 (Src homology region 2 domain-containing phosphatase-2), linking receptor tyrosine kinases, and the MAPK signaling pathway have been reported to increase in melanoma samples [35]. Specifically, within the melanoma microenvironment, Zhang et al. [36] reported that SHP2 positively regulates ERK and AKT signaling and, therefore, melanoma proliferation and survival. When SHP2 is targeted in vivo by an inhibitor such as 11a-1, melanoma xenograft tumor growth is suppressed. SHP2 is upstream of the alternate pathways, such as AKT and mTOR, and is employed by BRAFi–MEKi double-resistant melanoma samples, suggesting that SHP2 inhibition can effectively treat BRAFi and MEKi double-resistant cells.

While BRAF and MEK inhibition is a common tactic for treating melanoma, ERK-specific targeting has been rare despite its placement downstream of both BRAF and MEK, which has a more direct effect on the genetic and proliferative effects of the MAPK signaling pathway. Some researchers have developed ERK inhibitors (ERKis), such as SCH772984, which are highly selective and can be used as a viable supplement in combination with BRAFis and MEKis or replace them in subsequent acquired BRAFi–MEKi double resistance. Morris et al. [37] noted that SCH772984 effectively treated melanoma cells with and without BRAFi and MEKi resistance because the alternate pathways used in double resistance are often associated with ERK reactivation. However, if ERKi resistance develops, the resulting melanoma samples may prove to be even more challenging to treat due to the limited knowledge of alternate pathways subsequent to ERK.

An additional factor in BRAF mutation melanoma survival is the level of autophagy present in melanoma samples. Abnormal rates of autophagy and its related genes such as *Atg5* and *Sqstm1/p62* have been found within BRAF-mutant melanoma samples [38]. For example, although both genes are known to promote autophagy, Karras et al. [39] found *Atg5* to decrease with heterozygous deletions, while *Sqstm1/p62* expression was amplified in proliferative melanoma. However, the relationship between melanoma proliferation and autophagy is not solely based on genetics. Many factors, such as ER stress, can influence autophagy. Ma et al. [40] showed that the addition of BRAF inhibitors to melanoma cells caused an increase in autophagy due to the binding of mutant BRAF to the ER stress gatekeeper GRP78. Subsequent BRAF and autophagy inhibition via hydroxychloroquine caused a reduction in tumors in BRAFi-resistant cells. Other studies have noted that autophagy may be tumor-suppressive in the early stages of tumor development and then become tumorigenic in later stages [41].

Another approach that could be considered to improve the current state of BRAF and MEK inhibitory therapies is the use of predictive biomarkers to guide treatment decisions between patients [42]. For example, markers of tumor burden, such as serum lactate dehydrogenase levels and the number of organ sites with metastatic features, were correlated with better outcomes in PFS and overall survival (OS) in patients that received combination therapy of dabrafenib and trametinib [43]. The discovery of new oncogenic driver mutations as potential biomarkers may help predict responses for targeted therapies in certain patient populations.

### 2.2. Non-BRAF and MEK-Related Biomarkers

Melanomas with mutations in NRAS, the second most prevalent mutated proto-oncogene in melanoma, are characteristically more aggressive and susceptible to developing resistance to treatment when compared to BRAF mutants [2,5]. Unlike BRAF mutants, melanoma harboring an NRAS mutation alters GTP hydrolysis to consequently activate the MAPK, PI3K, and RAS-like protein guanine nucleotide exchange factor (GEF) signaling pathways [5]. In total, 90% of NRAS mutations in melanomas are NRAS^Q61R^ [44]. In these melanomas, MAPK is activated via CRAF, not BRAF, and these tumors also have aberrant cyclin-dependent kinase (CDK) 4/6 expression and hyperactivated PI3K/AKT signaling. Currently, the standard of care for NRAS-mutant patients involves anti-PD-1-based immunotherapy (see below) as the first line of treatment, with the second line of treatment including MEK and CTLA-4 inhibitors, albeit with varying degrees of success [45]. The lack of druggable pockets within RAS makes it hard to develop RAS-specific inhibitors [2], although recent structural studies of mutant KRAS offer promising new therapeutic strategies [46]. Another study using the FAK inhibitor defactinib, Del Mistro et al. [47] demonstrated that the inhibition of FAK can desensitize melanoma cells regardless of BRAF/NRAS mutational status. While the efficacy of defactinib is not certain, clinical trials are currently being conducted employing defactinib to treat metastatic melanoma and other cancers in a phase II trial (NCT04270417) [48].

Other non-V600 BRAF mutants, which have been reported in up to 14% of melanoma patients, are very rare, and therefore, little is known about their available treatment [49]. Since these melanomas can often activate the MAPK pathway through uncommon means, such as CRAF, pan-RAF inhibitors, like sorafenib and AZ628, have been explored on non-V600 BRAF-mutant melanoma samples [50]. Molnár et al. [50] reported that a combination of sorafenib or AZ628 and selumetinib, an MEK inhibitor, can effectively combat non-V600 BRAF-mutant melanoma in preclinical mouse models. Furthermore, recent clinical trials, such as the phase I trial (NCT01425008) by Rasco et al. [51], investigated the safety and antitumor activity of tovorafenib, another pan-RAF inhibitor and showed encouraging results. In all, these studies represent promising steps forward for the use of pan-RAF inhibitors on not only BRAF^V600E^ but also BRAF-wild type and CRAF-wild type melanoma patients.

CDK4/6 is another molecule involved in the development of melanoma [52]. The common role of CDK4/6 is to bind with Cyclin-D1, enter the nucleus, and hyperphosphorylate the retinoblastoma (RB) protein; this results in the release of the transcription factor E2F, which drives the transcription of genes for cell cycle progression. In melanoma, multiple mutations can affect CDK4/6 activity and increase proliferation by promoting the G1-S transition [53]. Interestingly, CDK4/6 can be activated beyond the MAPK pathway via the estrogen receptor (ER) signaling pathway, which is common in breast cancer [54,55]. Because CDK4/6 inhibitors have already been developed and effectively used to treat breast cancer tumors, clinicians are testing the efficacy of CDK4/6 inhibitors in combination with other inhibitors to combat melanoma. For example, Yoshida et al. [56] discovered that vemurafenib-resistant tumors were still sensitive to palbociclib (a CDK4/6 inhibitor). This has been further explored by Jost et al. [57], who found that palbociclib induced cell cycle arrest in melanoma cells when used in combination with radiotherapy. These findings have identified CDK4/6 as another potential path for treating patients with BRAFi- and MEKi-resistant melanoma.

AXL, a tyrosine kinase receptor, is another potential target due to its notable elevated expression in many cancers, including melanoma [29,58]. Once bound by a ligand, AXL will activate downstream signaling pathways that affect cell cycle progression, including the PI3K/AKT pathway. AXL is involved in many crucial roles in cancer development, including cell movement, immunosuppression, and epithelial–mesenchymal transitions (EMTs) [59]. AXL expression is also correlated with acquired MAPK resistance [29], including BRAFi/MEKi double resistance, and targeting AXL cooperatively inhibited tumor growth with BRAF/MEK inhibitors in patient-derived xenografts [60]. Recent in vitro studies have shown that the knockdown of AXL by siRNA or its inhibitor bemcentinib in melanoma cells decreased migration and invasion [58,61]. A phase II study (NCT02872259) comparing the efficacy of the standard melanoma treatment of dabrafenib, trametinib, and pembrolizumab with or without bemcentinib in patients with phase III or IV melanoma is currently ongoing [62].

Notably, most of the NF1 mutations associated with melanoma result in loss of function in the NF1 gene, classifying it as a tumor suppressor [63]. Approximately 12–18% of melanoma patients have mutations in NF1, which are most common in elderly and sun-exposed individuals. This protein has a GTPase-activating protein-related domain that negatively regulates RAS-GTP to the inactive RAS-GDP form, thus inhibiting the progression of the MAPK pathway. Current treatments of NF1-mutant melanomas rely on targeting other proteins, such as MEK and anti-PD-1 therapy [64,65].

Met (mesenchymal–epithelial transition factor receptor), along with its paracrine-induced ligand HGF (hepatocyte growth factor), is another signaling pathway that has been targeted for melanoma therapy [66,67]. Like with other kinase receptors, abnormal activities of Met and HGF have been associated with cancers such as melanoma. What is unique about Met in melanoma cell lines is that HGF is secreted via autocrine and paracrine signaling [68], often leading to a positive feedback loop of growth [69]. Drug inhibitors for the Met/HGF signaling pathway, such as crizotinib, tivantinib, and quercetin, are undergoing experimental trials with promising results [70]. For example, using tumor samples from melanoma patients, Das et al. [71] demonstrated that a combination of crizotinib and a tyrosine kinase inhibitor, afatinib, reduced melanoma tumor growth, regardless of BRAF/NRAS mutational status. Additionally, PHA665752, a drug that blocks MET phosphorylation, has shown to be effective in targeting NRAS-mutant cells during in vitro studies, indicating the broader effectiveness of Met inhibitors in treating various melanoma subtypes, not only the common BRAF^V600E^ mutant [72].

### 2.3. Melanoma-Stem-Cell (MSC)-Related Biomarkers

With recent advancements in our growing knowledge of stem cells, general biomarkers associated with melanoma stem cells (MSCs) have been elucidated, including CD133 [73], CD271 [74], and ABCB5 [75]. Targeting MSC biomarkers is expected to benefit patients with melanoma. For example, CD133, a critical biomarker of MSC to maintain stemness properties and drug resistance, is reported to be upregulated in melanoma and involved in tumor growth, angiogenesis, and metastasis via mechanisms of PI3K/AKT and MAPK activation [76], suggesting inhibition of PI3K/AKT and/or MAPK signaling pathways not only targets melanoma with BRAF and NRAS or other gene mutations but also battles MSCs [29,30,33,76]. Knockdown of CD133 expression in NRAS-mutant melanoma promoted cell apoptosis and improved trametinib efficacy in the NRAS-mutant cells [77]. A recent study reported that a vaccine against MSCs (CD44^+^CD133^+^ cells) stimulates immune response and inhibits melanoma growth and metastasis in vivo [78]. However, due to these biomarkers exhibiting other types of cancer as well as normal stem cells, studies should assess tolerability and efficacy.

### 2.4. Non-Genomic Biomarkers in the Melanoma Microenvironment

Beyond a high mutation rate, melanoma often rewires its metabolism program through non-genomic regulation to provide a favorable tumor microenvironment (TME) for supporting tumor cell growth and suppressing immune surveillance [11,12,79]. Targeting the vulnerabilities of metabolism may improve melanoma therapy. For example, NRAS-mutated melanoma cells reprogram a quiescent metabolic program to avoid MEK-inhibition-induced cell apoptosis [80] with increased reactive oxygen species (ROS) levels, making these cells highly sensitive to ROS induction. Thus, treatment with an ROS inducer and an MEK inhibitor inhibited tumor growth and metastasis [80]. Studies have shown that melanoma patients with high levels of lactate dehydrogenase (LDH) often have a worse prognosis and low response to checkpoint therapy, implicating that inhibition of LDH may provide an opportunity to modulate the TME favorably. Treating patient-derived melanoma with a lactate dehydrogenase A (LDHA) inhibitor, GSK2837808A, showed T-cell antitumor cytotoxicity. Recently, a compound AZD3965 developed to target MCT1 and MCT4 of LDH transporters is under investigation in a clinical trail (NCT01791595) [81]. Within the TME, fibroblasts also make functional shifts to cancer-associated fibroblasts (CAFs), which are known to support melanoma immune evasion and tumor growth via many proteins, including FBLN1 and COL5A1 [82]. Inhibiting the expression of FBLN1 and COL5A1 by mifepristone and dexamethasone drugs has potentially improved patient outcomes [82]. Furthermore, antibody–drug conjugate ABBV-085 against LRRC15 of the new CAF biomarker showed significant antitumor activity with minimal toxicity [83]. In addition to melanoma in the TME, circulating tumor cells (CTCs) are proposed to have dominant mitochondria-mediated oxidative phosphorylation (OXPHOS) [84], suggesting anti-OXPHOS may prevent melanoma metastasis.

## 3. Advances in Immunotherapy

### 3.1. Immune Checkpoint Inhibitors (ICIs)

Currently, the preference for the clinical treatment of advanced and high-risk, early-stage melanoma is ICI therapy [85]. This systemic therapy offers a unique advantage by not only inducing cancer cell eradication but also by extending survival through anti-cancer maintenance that improves overall survival with some first-line ICIs, making it the preferential treatment for most metastatic melanoma cases [86]. ICIs can ultimately block the tumor’s ability to escape the immune system.

The first immune checkpoint to be identified in treating cancer was cytotoxic T lymphocyte-associated protein 4 (CTLA-4), which competes with the costimulatory molecule CD28 for ligands CD80 and/or CD86 (collectively known as B7 ligands) [19,87]. In contrast to CD28, CTLA-4 has a greater binding affinity and avidity for these two ligands. The ensuing deprivation of costimulatory signals to T cells was eventually linked to the finding that anti-CTLA-4 antibodies result in tumor regression in preclinical mouse models [88]. Ipilimumab, tremelimumab, and BCD-145 are the three major anti-CTLA-4 human monoclonal antibodies for use against metastatic melanoma and are currently undergoing preclinical and clinical trials [19,89].

Secondly, programmed cell death protein 1 (PD-1) and its ligand programmed cell death ligand 1 (PD-L1) are other immune checkpoints for standard melanoma immunotherapy. CD8^+^ exhausted T (T_EX_) cells lose effector function during the antigen stimulation process for malignancies. By blocking PD-1 and, by extension, PD-L1, the effector functions of CD8^+^ T cells can be restored, resulting in improved tumor control [87]. In 2014, anti-PD-1 antibodies pembrolizumab and nivolumab were granted FDA approval for clinical use against metastatic melanoma. These ICIs have been joined by several other anti-PD-1/PD-L1 antibodies, including avelumab, durvalumab, cemiplimab, atezolizumab, and cosibelimab [19]. Additionally, while ICIs were initially approved for use as monotherapies, recent evidence has shown that combining multiple immunotherapies can result in an augmented anti-tumor response and a greater degree of long-term efficacy. Thus, there has been increasing interest in a combined anti-PD-1/PD-L1 and anti-CTLA-4 checkpoint blockade for the treatment of melanoma [90]. For example, a phase Ib/2 trial (NCT02535078) found that the combination of tebentafusp with durvalumab and/or tremelimumab is effective in treating advanced or metastatic melanoma [89]. The current landscape regarding anti-CTLA-4 and anti-PD-1/PD-L1 therapy focuses on optimizing dosages and reducing associated toxicity events, which are not insignificant in the clinic. A significant phase III trial testing the combination of ipilimumab and nivolumab indicated that it was effective in treating both advanced melanoma and melanoma that has metastasized to the brain [91]. Interestingly, patients in this study with BRAF mutations fared better than those with wild-type BRAF. van Zeijl et al. [91] report that this was most likely due to BRAF-mutant patients also receiving BRAFi and MEKi (dabrafenib and trametinib, respectively) treatment.

Success surrounding the use of the aforementioned ICIs has led to the continuing identification of novel immune checkpoint molecules. Lymphocyte activation gene-3 (LAG-3, also named CD223) is a surface inhibitory receptor with structural similarities to CD4 and is a promising new target for immune checkpoint blockade [9,87,92]. The suppressive function of LAG-3 is contributed to by its constitutive overexpression on regulatory T cells (T_REGS_) [92]. At present, relatlimab is the most developed anti-LAG-3 antibody, and a randomized double-blind phase II/III trial (NCT03470922) is currently investigating its effectiveness in combination with anti-PD-1 antibodies, principally nivolumab, in several tumor models, including melanoma [93]. According to the study’s findings, relatlimab paired with nivolumab results in improved PFS compared to nivolumab monotherapy [94]. It remains unclear which combination ICI therapy has better antitumor efficacy while simultaneously decreasing toxicity levels. Further clinical trials, such as the phase I trial (NCT04140500) investigating the effect of a bispecific anti-PD-1 and anti-LAG-3 antibody (RO7247669) on solid tumors, may validate the above conclusions and expand the treatment options for patients with melanoma [95,96].

While the management of advanced solid tumors has been significantly impacted by the increasing availability of ICIs [97], many patients either do not respond to immunotherapy or experience adverse outcomes. Thus, it is also important to explore other potential biomarkers and develop further combination treatments that may improve response rates and outcomes [97]. B7 homolog 3 protein (B7-H3) is currently being explored as a target for next-generation cancer immunotherapy, entering many clinical trials as a therapeutic target [98]. B7-H3 has been found to be overexpressed in many solid cancers (including melanoma) and is a biomarker of disease severity and recurrence [99]. MGC018, a duocarmycin-based antibody–drug conjugate targeting B7-H3, has displayed potential antitumor activity in preclinical melanoma models with a favorable pharmacokinetic safety profile [99]. In the phase I/II clinical trial, the dual blockade of B7-H3 and PD-1 with enoblituzumab and pembrolizumab has demonstrated acceptable safety and antitumor activity in patients with solid tumors [97]. However, there has been a limited response in patients with cutaneous melanoma, with only one out of thirteen patients exhibiting a partial response.

T-cell immunoreceptors with immunoglobulin and the immunoreceptor tyrosine-based inhibition motif domain (TIGIT) and its ligand CD155 are also being explored as a new immune checkpoint target for their role in delivery inhibition signals to T cells, NK cells, and regulatory T cells [100]. TIGIT expression can be closely associated with melanoma occurrence, development, and prognosis; consequently, decreased TIGIT expression is associated with inhibited tumor growth in melanoma patients. The TIGIT/CD155 axis has also been implicated in mediating resistance to ICIs, where TIGIT blockade or CD155 deletion in activated T cells has aided in overcoming ICI resistance [101]. CD96, a receptor protein that can regulate NK cell effector function and metastasis, is also of note as it can interact with CD155, and blocking CD96 can suppress primary tumor growth in mouse tumor models [102]. The addition of anti-CD96 in combination with anti-PD-1, anti-CTLA-4, anti-TIGIT, or doxorubicin chemotherapy resulted in superior antitumor responses by enhancing T-cell activity and suppressing tumor growth [102,103].

T-cell immunoglobulin domain and mucin domain-3 (TIM-3) is another biomarker of interest, as its ligand (Galectin-9), along with PD-L1, are both upregulated during tumor progression [104]. Prokopi et al. [104] found that boosting DC in combination with anti-PD-1 and anti-TIM-3 therapy improved T-cell function within tumors and delayed tumor growth. A phase I/Ib clinical trial has demonstrated that the combination treatment of sabatolimab (MBG453) and spartalizumab, monoclonal antibodies that can bind to TIM-3 and PD-1, respectively, can be well tolerated and show preliminary signs of antitumor activity in advanced solid tumors, including one patient with malignant perianal melanoma [105]. Additionally, a novel melanoma-stem-cell vaccine has been developed that can suppress the expression of CTLA-4, PD-1, and TIM-3 and delay the progression of melanoma by inducing antitumor immune responses [78].

Human leukocyte antigen G (HLA-G) is also of note as it is one of the genes found to be commonly upregulated in premetastatic brain-metastasis-initiating cells (BMICs) [106]. HLA-G was found to function in an HLA-G/SPAG9/STAT3 axis that promotes the establishment of brain metastatic lesions. Overall, identifying clinically relevant biomarkers can inform the development of next-generation immunotherapies [106]. Characterizing a patient’s relevant biomarkers can reveal the optimal treatment strategy for each patient.

### 3.2. Adoptive Cellular Therapy (ACT)

In the scope of advanced cutaneous melanoma, adoptive cellular therapy (ACT) is a relatively new treatment approach that is geared toward treatment-refractory patients who have exhausted all approved therapy options [13,107,108,109]. ACT is a subsection of immunotherapy that relies on the internal and external manipulation of patients’ immune systems to construct a personalized approach to treating metastatic or unresectable solid tumors [13,108]. In brief, endogenous immune (T or NK) cells are isolated from the patient, selected, and expanded ex vivo before reintroduction into the patient [108]. The current state of the ACT field can be broken down between the growing developments of two major techniques: tumor-infiltrating lymphocyte (TIL) and chimeric antigen receptor (CAR-T) therapies. One of the significant differences between TIL-based ACT and CAR-T therapy is the type of cells that are ultimately reintroduced to the patient. TILs express unmodified endogenous T-cell receptors (TCRs), while CAR-T therapy uses TCRs that have been synthetically modified to recognize a specific antigen [13,108].

#### 3.2.1. Tumor-Infiltrating Lymphocytes (TILs)

The transformation from normal to malignant cells is facilitated by a multitude of genetic mutations and changes to the TME that result in heterogeneity differences between the tumors of patients, even in those diagnosed with similar malignancies [108]. Developing personalized therapies that account for these tumor-specific characteristics is of utmost importance. Recently, autologous tumor-infiltrating lymphocytes (TILs), or TIL-based ACT, have been developed based on this concept. TIL-based ACT follows a three-step workflow: (i) isolation of TILs from tumor excision, (ii) rapid ex vivo expansion of TILs, and (iii) infusion of TILs back into the lymphodepleted patient during hospitalization [13,108].

While TILs can recognize many targets in cancer and TIL-based therapy remains the preferential treatment option for most metastatic melanoma cases, some significant limitations prevent TIL-based ACT from expanding as a widely used treatment option for patients. These challenges include the manufacturing requirements of TILs, treatment-related toxicity events, and treatment resistance. First, TIL production is both labor-intensive and complex, and thus only available primarily to well-funded medical centers that can accommodate the necessary technology to handle the TIL workflow and potential treatment-emergent adverse events (TEAs) that could occur during patient hospitalization. It is imperative to centrally expand the manufacturing process of TIL products as it could allow for a widespread application of TIL-based ACT that is both cost-effective and accessible, such as Iovance Biotherapeutics and their development of a central manufacturing facility that produces Lifileucel, a cryopreserved, autologous TIL product [108,109]. Encouragingly, the FDA has recently approved lifileucel (Amtagvi) for advanced melanoma.

Second, reinfusion also requires the patient to undergo a pre-conditioning regimen—the side effects of which make up most reported treatment-related toxicities. These toxicities can either be cytokine-related toxicities, resulting from the high levels of IL-2 frequently given with TIL therapy to enhance the lymphocytes’ antitumor activity, or rare autoimmune-related toxicities, commonly caused by the non-specific expression of tumor-associated antigens on non-cancer cells that can become targeted by reintroduced lymphocytes [110,111]. This preparative lymphodepleting regimen, comprising a combination of cyclophosphamide and fludarabine, has been shown to increase the effectiveness of TIL-based ACT, although the cellular mechanisms are not fully understood at this time and the tradeoff in terms of severe toxicities is substantial [13].

Finally, the use of TILs, as in many cases, is vulnerable to resistance. Both innate and acquired resistance are prevalent, where innate resistance refers to observed unresponsiveness following the initial therapy administration, and acquired resistance refers to a developed resistance that presents itself after a patient’s previous positive response [108]. The mechanisms resulting in these resistance types can be broken down into four main distinctions: (1) curated T cells fail to recognize tumor cells effectively, (2) interference from immunosuppressive cells in the TME, (3) TME-driven T-cell dysfunction and/or exhaustion, and (4) restrictions in T-cell migration to the tumor [13,108]. While the mechanisms underlying these resistance phenomena are becoming better understood, work remains to be done to further optimize the curation of TILs. Promisingly, current work has focused on combining TIL therapy with other therapies, such as the prospective randomized phase II trial (NCT02621021) currently in progress that seeks to understand if the addition of pembrolizumab with TIL/IL-2 therapy can improve response rates in metastatic melanoma patients [112].

#### 3.2.2. Chimeric Antigen Receptor (CAR) T-Cell Therapy

Chimeric antigen receptor (CAR) T-cell therapy, one of the first personalized techniques commercially available to the clinical population, involves three main stages to generate clinically utilizable CAR T cells: selection, expansion, and harvesting [113]. During the selection stage, T cells collected from the patient via leukapheresis undergo monocyte elutriation to remove other cell types (e.g., myeloid, natural killer, erythroid, and malignant cells) and allow for efficient extraction and isolation [113]. The selected T-cell product is then activated and genetically transduced with a viral vector that encodes for the tumor-antigen-specific CAR construct. The T-cell product is then expanded ex vivo to generate a high yield of engineered cells before being harvested to form the final CAR-T cell product that is reinfused back into the patient [113]. Like TIL-based ACT, patients must receive conditioning chemotherapy to deplete autologous lymphocytes and immunosuppressive cells [114]. Two CAR-T cell constructs for the CD19 protein—tisagenlecleucel (Kymriah, Novartis, Basel, Switzerland) and axicabtagene ciloleucel (Yescarta, Kite, Foster City, CA, USA)—have recently been approved for the treatment of B cell lymphoma. Axicabtagene was noted to have a slightly higher 12-month overall survival than tisagenlecleucel (51% and 47%, respectively), but they both had similar efficacy [115].

CAR-T cell research currently focuses on translating this success to solid cancer tumors. CAR constructs can recognize whole surface proteins on cancer cells without relying on the presentation of the major histocompatibility complex (MHC) and antigen processing [1]. The ability of these constructs to function in a non-MHC-restricted manner makes this subset of adoptive cell therapy a promising option for the immunogenic features of melanoma [116]. CAR constructs express chimeric antigen receptors with three main domains of interest: extracellular, transmembrane, and intracellular. The extracellular domain houses the single-chain variable fragment (scFv), which is composed of an antibody-variable heavy chain (V_H_) and an antibody-variable light chain (V_L_) that have been fused together by a peptide linker [116]. This scFv domain is further linked to the intracellular CD3ζ domain through the transmembrane domain. CAR constructs are classified into generations, where first-generation constructs contain only the CD3ζdomain while ensuing generations have increasing numbers of additional co-stimulatory molecules (i.e., CD28, 4-1BB, OX-40) [1]. While CAR-T cell therapy has shown promising results for hematological malignancies [1,116], initial attempts at using CAR-T cell therapy to treat other cancers have not been as successful [117,118,119]. Specifically in metastatic melanoma, challenges include (1) selecting an optimal antigen target and (2) the influence of the immunosuppressive TME [116].

First, selecting an optimal antigen target has the dual goal of inducing an anti-tumor immune response while producing the lowest amount of off-target toxicity and immune side effects for patients. This off-target toxicity is commonly observed when the target antigen is expressed in both healthy tissue and malignant tumor tissue; consequently, multiple targets whose expression is limited to malignant tissues alone have been identified [12,120,121]. For example, CD248 is a type I transmembrane glycoprotein that is either not expressed or minimally expressed in healthy tissues [1,121]. Interestingly, CD248 has been reported to play a role in tumor vasculature and was expressed in 86% of metastatic melanoma samples analyzed by tumor microarrays [120]. Another potential target is chondroitin sulfate proteoglycan 4 (CSPG4), also known as melanoma chondroitin sulfate proteoglycan (MSCP), which is expressed in 90% of melanomas as well as in sarcomas and gliomas but rarely expressed in healthy tissues [122,123]. Whether CD248 and CSPG4 could be optimal antigen targets for metastatic melanoma in CAR-T cell therapy remains to be validated. Thus, future preclinical investigations for potential use in a clinical setting remain necessary to select the proper target antigens. Immune side effects of CAR-T cell therapies include cytokine release syndrome (CRS), encephalopathy syndrome (CRES), and immune effector cell-associated neurotoxicity (ICANS), which—with appropriate treatment and observation by clinicians—can be minimized and even reversible [124].

Second, the TME refers to the complex environment surrounding each cancer cell. Several properties of the TME (e.g., extracellular matrix, cytokines, growth factors, hypoxic conditions, common cell types such as fibroblast and immune cells) are unfavorable for CAR-T cell therapy as they can reduce the potency of the anti-tumor response and allow for continued tumor growth and invasion [1,125,126]. To combat this, significant attempts have been made to modify the CAR constructs in solid tumors like melanoma by blocking the activation of inhibitory immune checkpoint receptors on T cells, with the most common of these receptors being PD-1 and CLTA-4. Successful attempts include stable knockouts of the inhibitory receptors via CRISPR/Cas-9 and the development of CAR-T cells that are capable of constitutively secreting immune checkpoint inhibitors [127,128,129]. For example, Marotte et al. [130] designed PD-1 knockout TCR-engineered T cells specific for the Melan-A antigen. Their findings revealed these engineered T cells garnered higher anti-tumor efficacy and delayed PD-L1-positive melanoma tumor progression in mouse models. Given the already established role of immune checkpoint blockade as standard therapy in advanced melanoma cases, there is the potential for combination therapy that pairs anti-PD-1 and anti-CTLA-4 antibodies with ACT to produce better clinical outcomes; some studies with lymphoma and malignant plural disease patients have already begun to move in this direction [131,132].

CAR-T cell therapy brings an innovative technique to the multifaceted space of cancer immunotherapy. Still, various barriers continue to prevent this from becoming a standard therapy in the treatment of melanoma and other solid tumors. The use of newly engineered CAR-T cells, the discovery of suitable target antigens, and combination therapy techniques to alter the TME aim to eliminate these obstacles and guide the future clinical use of CAR-T cell therapy in melanoma. Additionally, natural killer cells with chimeric antigen receptors (CAR-NK cells) are a recent development in immunotherapy [11,12]. Unlike CAR-T cell therapy’s high immunologic systemic toxicity, CAR-NK cell therapy displays lower toxicity because it has a shorter in vivo duration [133]. Uniquely, NK cells are dependent on a balance between activating and inhibitory germline-encoded signals which are not susceptible to downregulation in cancerous cells [12]. Furthermore, CAR-NK therapy can be allogeneic and therefore safer and manufactured “off-the-shelf”, indicating a high potential for successful future treatments [134]. For example, a phase I clinical trial using CAR-NK cell therapy against anti-PDL1/MUC1, a glycoprotein known to be overexpressed in melanoma and promote metastasis, was shown to display a stable response in a majority of patients with a range of solid tumors [135,136]. This may indicate a path forward for CAR-NK therapy’s effectiveness as a personalized treatment for patients with melanoma.

### 3.3. Vaccine Development

Ideally, like the prevention of infectious diseases, administering a cancer vaccine could help the immune system detect and eliminate tumors. Unfortunately, hundreds of attempts have not made significant improvements in patients’ health. However, one notable study reported that personalized vaccines for melanoma targeting mutated proteins using mRNA increased T-cell infiltration that led to antitumor activity across patients, providing optimism for the future success of this approach [137]. Vaccines can potentially create a targeted and tumor-specific immune response whose long-term memory may aid in cases of subsequent metastasis for treatment-refractory patients, especially in melanoma with high immunogenicity. The combination of vaccines with other immunotherapies also offers greater tumor control. Regardless of vaccine type and antigen or adjuvant choice, the backbone of vaccine development is the injection of tumor antigens in an immunostimulatory space to prime tumor-specific T cells or induce antibodies while breaking tolerance to self-antigens and causing tumor cell death [138]. Currently, vaccines developed for melanoma treatment have been whole-cell vaccines, peptide-based vaccines, dendritic cell (DC) vaccines, ganglioside vaccines, DNA vaccines, and RNA vaccines [139,140].

#### 3.3.1. Whole-Cell Vaccines

Whole-cell vaccines can be split into two subtypes: autologous and allogeneic tumor cell vaccines [140]. Autologous whole-tumor cell vaccines are manufactured using either excised tumor cells or from autologous tissue culture that has undergone ex vivo irradiation to eliminate the ability to replicate [138,139]. Although this autologous subtype is patient-specific because the components are derived from the recipient, this technique lacks broad applicability. Additionally, the process is both time- and labor-intensive, requiring an adequate amount of tumor tissue from each patient, and attempts at quality vaccine preparation have been met with high degrees of failure [141].

Allogeneic vaccines derived from whole-tumor cells refer to those generated from melanoma tumor cells derived from patients who are not the intended vaccine recipient and may contain more than one tumor cell line to augment the antigen expression profile [138,139]. Like autologous whole-cell vaccines, the tissue requires prior irradiation to be rendered replication-deficient. Advantages include an expanded range of available tumor antigens, broad applicability to multiple patients, and lack of requirement for a patient’s specific tumor tissue. However, there is the possibility of a lack of patient specificity. The response of allogeneic whole-cell vaccines relies on how well the tumor cells in the vaccine match the tumor cells of the treated patient. Two well-known examples of allogeneic whole-cell vaccines are Canvaxin™ (CancerVax Corporation, Carlsbad, CA, USA) and Melacine^®^ (Corixa Corporation, Seattle, WA, USA) [138,139]. Canvaxin is composed of three melanoma cell lines, boasting over 20 melanoma-associated tumor antigens, and is administered with Bacillus Calmette–Guerin (BCG) as an immunoadjuvant; unfortunately, multiple phase III trials failed to reveal the benefit of Canvaxin over the investigated placebo [142]. Melacine comprises two melanoma cell lines that are paired with immunoadjuvant “detoxified Freund’s adjuvant” (DETOX); moreover, like Canvaxin, Melacine failed to demonstrate a significant benefit in disease-free survival once reaching phase III trial stage [138,139,143].

#### 3.3.2. DNA Vaccines

DNA vaccines comprise naked DNA expression plasmids that possess a gene encoding for the target antigen(s) from melanoma tumor cells [144]. Therefore, DNA vaccines immunize patients using plasmid-encoding antigens rather than with the antigen. The administration is commonly given through parenteral routes (i.e., intramuscular, subcutaneous, transdermal, or intradermal). However, some attention has been recently given to mucosal routes (i.e., intranasal, vaginal, and oral) due to the advantage of generating local immunity at specific sites [144,145]. DNA vaccines are traditionally low-cost, highly stable, and less laborious when compared to other vaccine therapy options. However, disadvantages include the low immunogenicity of plasmid DNA, the possibility of virus reversion, and tolerance against autoantigens if the vaccine is administered without an immunoadjuvant [138,144].

Tyrosinase, a glycoprotein that is necessary for melanin synthesis and can promote an immune response against melanogenesis-related antigens, has been a target of interest for DNA vaccines. Some studies have reported that administering tyrosinase could induce antigen-specific T-cell responses, and several DNA vaccines based on the tyrosinase antigen have been developed [144,146]. For example, the Oncept melanoma vaccine—a DNA vaccine that is used to treat melanoma in canines—uses human-DNA-encoding tyrosinase to elicit an immune response in dogs; however, while the vaccine appears to be safe, its efficacy is limited [147].

Additionally, the melanoma antigen-1 (MAGE-A1) family is known to have increased expression in human cancer types, including melanoma, and has also become a target of interest for DNA vaccines. Duperret et al. [148] found that targeting any member of the MAGE-A family, not just the commonly upregulated MAGE-A3, via DNA vaccination effectively produced a robust immune response that slowed tumor development and prolonged the median survival of mice. By targeting a wider range of proteins, the treatment is more generalized to the heterogeneity of the TME while reducing toxicity. Although some progress has been made in the treatment of melanoma with DNA vaccines at the preclinical animal model stage, more clinical studies are needed to validate DNA vaccine efficacy.

#### 3.3.3. RNA Vaccines

Tumor-associated antigens (TAAs)—commonly cancer germline antigens or lineage-specific differentiation markers—have become the core of cancer immunotherapy and are attractive targets for RNA vaccines because they can cause cells to synthesize TAAs that are recognized by T cells and subsequently trigger a targeted immune response [140,149]. In the past however, this strategy has been clinically ineffective in many vaccine trials due to a central T-cell tolerance to TAAs [150], with this ineffectiveness especially prevalent in advanced-stage patients with lower mutational burdens [151].

Sahin et al. [149] report a novel intravenously administered nanoparticulate-liposomal RNA (RNA-LPX) vaccine known as melanoma FixVac (BNT111) that has been introduced in the first-in-human phase I trial (Lipo-MERIT, NCT02410733) using an RNA vaccine. The RNA-LPX vaccine contains optimized RNA targeting immature DC in lymphoid tissues. It is composed of four TAAs that are present on both MHC class I and class II molecules—NY-ESO-1, MAGE-A3, tyrosinase, and TPTE—which express at restricted levels in normal tissues but have a high prevalence and immunogenicity in melanoma. Preliminary results have shown that patients experienced increased spleen metabolic activity, indicating the TLR activation of lymphoid-tissue-resident immune cells. ELISpot analyses confirmed that most patients had a strong T-cell response to at least one of the four TAAs, mostly being CD4^+^ or a combination of CD4^+^ and CD8^+^ T-cell de novo responses [149,150]. These effector T cells remained stable in cohorts that received a continuing vaccine dosage, and long-term memory T cells persisted in patients who did not receive any further vaccination beyond the initial doses. Interestingly, some of the patients who had prior anti-PD1 therapy failure showed signs of tumor regression following vaccination doses and afterward responded to an ensuing round of anti-PD1 therapy [149,150]. Taken as a whole, these study results mark the beginning of what may be a promising therapeutic option for a vaccine targeting TAAs, especially when employed in combination with other immunotherapies in patients with lower mutational tumor burdens and previously treatment-refractory tumors. For example, the combination of the individualized neoantigen mRNA vaccine mRNA-4157 (V940) with pembrolizumab showed longer recurrence-free survival with a manageable safety then pembrolizumab monotherapy in resected melanomas [152].

At its current stage, vaccine therapy is not considered a standard treatment option for advanced cutaneous melanoma. Vaccines must show proven clinical efficacy against melanoma to bridge the gap from experimental therapy to standard treatment. Factors that need a more thorough investigation include optimal timing for the start of vaccine therapy and the type of adjuvant that may be considered adequate. It is largely agreed upon that the success of vaccine therapy for melanoma patients will rely on a multimodal combined approach whose actual clinical effect is yet to be elucidated.

## 4. Thinking Innovatively—Where to Go Next?

Although there are multiple treatment options for melanoma, significant barriers still hinder the survival rate of melanoma patients due to the nature of its various mutations and heterogeneity. Thus, it is important to consider how further research may expand on prevention, early diagnosis, disease prediction, and advancing personalized options.

### 4.1. scSeq Techniques

Single-cell sequencing (scSeq) techniques are a new and increasingly popular tool for identifying biomarkers in specific cell types. This technique involves isolating individual cells and analyzing the gene expression of each cell [153]. This information is especially helpful for analyzing melanoma samples due to their high tumor heterogeneity [154].

With scSeq, each patient cell can be analyzed to identify seemingly minute differences between tumor cells that can identify potentially more efficient molecular targets. More specific targets can be identified with this extensive and detailed analysis of cells to limit unnecessary damage and toxicity. However, as with many new technologies, greater specificity also corresponds with a high price and longer analyzing times. Additionally, as scSeq requires isolated cells, procedures to disassociate cells from each other remain challenging. However, as scSeq continues to be developed and refined, more efficient and cheaper options should become available to make this analytical method accessible to all patients beyond highly funded research institutions to minimize socioeconomic-based disparities [155]. Many also predict that the emerging Human Cell Atlas project, a common database of all cell types, will help better identify mutant and/or tumorigenic cells. Studies, such as Davidson et al. [156], have used single-cell RNA-sequencing (scRNA-seq) to further define the melanoma TME landscape and serve as a resource to identify drug candidates in a manner that other researchers can employ. Furthermore, Ho et al. [157] used scRNA-seq to analyze melanoma patient samples and identify the role of CD58 in tumor cell immune evasion. They used samples of patients before and early on in their treatment plans of nivolumab with or without ipilimumab to identify the expression of CD58 in each patient’s cells. Utilizing scRNA-seq, Ho et al. discovered that the loss of CD58 confers cancer immune evasion in melanoma cells and that higher expression of CD58 is associated with anti-tumoral immunity. With this expanding database and potential applications, scSeq and its related exploratory data could be leveraged during therapeutic development. Moreover, further innovations will be needed for it to become more widely utilized [158].

### 4.2. AI and ML Development

More recently, artificial intelligence (AI) and machine learning (ML) are gaining attention in the field of oncology [159]. AI translates human problem-solving and comprehension skills to computers and can use ML to learn how to analyze and distill large amounts of data in less time than humans [160]. Deep learning (DL), a subset of ML, can also simulate human neural networks in order for machines to understand data automatically similarly to how humans instantaneously process sensory images to see the world.

AI/ML approaches comprise aspects that can be qualitatively as well as quantitatively superior to human analysis. In oncology, these technologies could allow clinicians to make precision-based predictions, diagnoses, and treatment decisions solely from analyzing patient data. Additionally, these technologies have the potential to improve accuracy, minimize patient sample volume collection, and detect melanoma and metastasis progression earlier [160,161]. For example, Marchetti et al. [162] demonstrated the use of an AI algorithm (ADAE) to analyze dermatoscopy images of skin lesions and subsequently predict melanoma risk. It was found that dermatologists had a significant improvement in their ability to assess melanoma risk after ADAE exposure.

With the high amounts of cellular heterogeneity in melanoma and a unique presentation in each patient, AI can be a necessary tool for clinicians to quickly analyze vast and complex information. Interestingly, the application of AI/ML/DL to existing patient data has created an improved, noninvasive method for predicting patients’ intracranial BRAF^V600E^ mutational status [163]. Moreover, using radiometric imaging data of patient samples, AI was able to better predict future disease progression and pembrolizumab effectiveness on early-stage melanoma samples from their baseline CT images than the standard clinician-based prediction method [164]. As a result, treatment plans can be further specified to target AI-predicted biomarkers and reduce “trial-and-error” drug therapies and resistance.

In summary, AI is a highly useful tool that can extend clinicians’ knowledge and scope to give patients a higher number of accurate precision medicine treatment options. This can result in earlier diagnosis, more precise treatment plans, and better overall outcomes and quality of life for cancer patients.

### 4.3. AAV-Mediated Gene Delivery System for Targeting Melanoma (CRISPR-Based (AAV))

Since cancer is commonly developed from genetic mutations, gene editing is an area of medicine with high potential for it. Gene editing technology using the Clustered regularly interspersed short-palindromic repeat (CRISPR)-Cas 9 system is a recent development for treating many diseases, with the first human clinical trials conducted in 2016 [165]. The CRISPR-Cas9 system involves two main components: guide RNA sequences that bind to the target gene with high specificity and the Cas9 endonuclease that allows for genome modifications by causing a double-stranded DNA break [166]. Ideally, through the CRISPR-Cas9 system, scientists and clinicians aim to restore a patient’s mutated cancerous DNA to a natural, non-tumorigenic state by altering the mutated gene, editing the mutated gene to the normal gene, or knocking out an amplificated oncogene [167].

One of the biggest hurdles to the use of CRISPR-Cas9 as a treatment is its delivery to patients. Recent studies have demonstrated that an adeno-associated virus (AAV) system could overcome this barrier [168]. AAV is a small, enveloped virus that can pack up to 5.0 kb of single-stranded DNA (ssDNA). With AAV’s inverted terminal repeats (ITRs) of 0.3 kb and the commonly used Cas9 consisting of 4.2 kb DNA, less than 0.5 kb of space is left for gene regulatory elements that guide gene editing. This limited available space causes larger gene targets of CRSIPR-Cas9 to be less commonly used than smaller cancerous gene targets [169]. Because of this, variables such as tissue specificity, off-target editing, and inducible expression are more difficult to control. To account for this, dual-vector delivery methods are being investigated in which two different AAV vectors are used, but this can reduce overall efficiency [169]. Additionally, smaller-sized Cas9 proteins, such as Cas9 from *Staphylococcus aureus*, are also being investigated as an alternative [170]. Fortunately, the use of AAV in vivo for smaller-sized melanoma-associated targets, like the proteins sBTLA+HSP70 for metastatic melanoma and the GM3(Neu5Gc) ganglioside for melanoma and breast cancer, has proven successful for anti-tumor activity in mouse models, indicating potential future translational and clinical success [171,172].

The clinical application of AAV vectors for CRISPR has been approved by employing AAV-CRISPR delivery directly to the eye to target the *CEP290* gene containing the mutation for blindness [173]. Many other CRISPR treatment protocols should start to gain clinical approval, suggesting that they will be implemented as a reliable treatment option for melanoma mutations—such as BRAF^V600E^—in the future.

### 4.4. Oncolytic Therapy Using Microorganisms (T-VEC)

#### 4.4.1. Bacteria

The hypoxic and necrotic regions that arise within the TME have proven to be a barrier to many treatments [174]. These regions are often poorly accessible to systemically delivered therapies. Low oxygen levels can reduce the efficacy of certain treatments and can affect the function of immune cells in vivo. However, therapy involving live tumor-targeting bacteria may present a unique option in overcoming these obstacles due to their ability to thrive and colonize within these niches. Historical evidence has shown that bacterial infections could induce anti-tumor responses, but this has only recently been pursued due to the current advances in genetic engineering for creating safer, attenuated strains of bacteria [174]. Because they have a high affinity for hypoxic and necrotic cell environments, bacteria could be used to deliver cytotoxic agents, prodrug-converting enzymes, and immunomodulators directly to tumor nodes in order to decrease immunosuppression, improve tumor-targeting specificity that decreases toxicity, and disrupt the tumor vasculature [175]. Bacteria may also induce an immune response that activates specific types of host immune cells—such as T cells and inflammatory cytokines (e.g., IL-1β, GM-CSF and TNF-α)—in order to recognize cancer cells as antigens, mark them for destruction, increase inflammation, and promote antitumor activity [176]. Unfortunately, this therapy still requires further research due to complications with translating in vivo models to human studies and health concerns regarding the use of potentially infectious bacteria populations. Wang et al. [177] note that some bacteria are tumor-associated and killing these bacteria in cancer mouse models improved immune recognition of tumor cells via the release of cancer-specific microbial neoantigens. However, even with the few clinical studies, such as the bacillus BCG administration of Canvaxin mentioned previously, this method can be best utilized in combination with other therapies, such as radiation or ICIs [178]. This was recently shown in mouse models by Chen et al. [179], who demonstrated that modified *Staphylococcus epidermidis* in combination with ICIs can reduce growth in localized and metastatic melanoma tumors.

#### 4.4.2. Viruses

Direct intralesional cancer immunotherapy is another treatment method that has been explored over the years, with a goal of inducing an effective control of disease in the injected lesions while also triggering a systemic immunological response [180]. Talimogene laherparepvec (T-VEC) marks the first oncolytic viral immunotherapy that has successfully gained FDA approval for the localized treatment of recurrent metastatic melanoma after surgery [181]. T-VEC is constituted by a genetically modified herpes simplex virus type I (HSV-1) that selectively replicates in tumor cells and transfects them with a granulocyte–macrophage colony-stimulating factor (GM-CSF) encoding plasmid, resulting in increased concentrations of GM-CSF in the TME. Locally, T-VEC causes the infected tumor cell to undergo increased antigen presentation for the recruitment of immune cells, causing lysis and a systemic polyclonal antitumor response [181,182]. T-VEC has been confirmed by pre-clinical studies to preferentially infect melanoma cells; additionally, clinical trials have demonstrated that it has promising efficacy in both monotherapy and in combination with ICIs [10,180]. When used in combination therapies, T-VEC may potentially improve the efficacy of ICIs due to its antitumor effects on the TME. This potential improvement in efficacy further supports the theory that combining immunotherapies with complementary modes of action may augment antitumor responses [183,184]. Studies remain ongoing to confirm the feasibility and efficacy of these combination strategies while producing low levels of clinical side effects [185].

## 5. Conclusions

**Figure 3:**
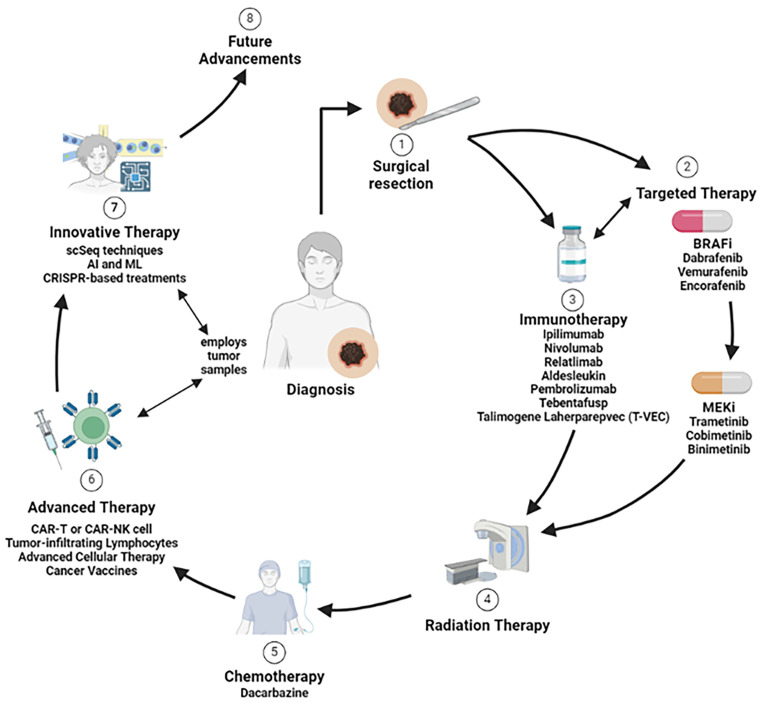
Optimization and personalization of treatment strategy for melanoma therapy. Due to differences in mutational status and other genetic considerations that drive a patient’s cancer specificity, there is not a “one-size-fits-all” solution for cancer treatment. The treatment options for melanoma include surgical resection, targeted therapy (e.g., BRAFis and MEKis), immunotherapy (e.g., anti-CTLA-4 and anti-PD-L1), radiation therapy, chemotherapy, advanced immunotherapy (e.g., CAR-T, CAR-NK, TILs, ACT), and innovative approaches that can be personalized to the patient’s disease. The ideal approach will depend on the mutational status and other genetic considerations that can drive a patient’s melanoma progression.

Due to differences in mutational status, other genetic and non-genetic considerations that drive a patient’s cancer specificity, there is not a “one-size-fits-all” solution for cancer treatment (Figure 3 and Table 1). Notably, melanoma is characterized by a higher rate of multiple gene mutations with strong intra-tumor and inter-tumor molecular heterogeneity, which makes treatment challenging. Individualized approaches are needed to determine which treatment or combination of strategies is best for each patient. Recent advances in translational treatment options, such as CAR-T cell therapy and vaccine development, are beginning to address this need for individualized therapy options. Still, more development is necessary to apply this universally to patient populations. Additionally, with emerging technologies, such as AI and CRISPR-Cas9, the coming decade of melanoma treatment research holds great promise in individualizing treatment options to improve melanoma patient outcomes and survival.

## Figures and Tables

**Table 1:** Current clinical and pre-clinical drugs for treatment of melanoma.

Drug Name	Target	Status	Notes	Reference
Vemurafenib	Binds to one of the ATP-binding sites of B-RAF	FDA Approved, 2011	Approved in Combination with Cobimetinib in 2015	[16]
Dabrafenib	Is an ATP-competitive inhibitor in a ATP-binding site of B-RAF	FDA Approved, 2013	Approved with Trametinib in 2022	[16,51]
Encorafenib	Binds to B-RAF and other kinases including CRAF and JNK1	FDA Approved, 2018	Approved in combination with Binimetinib in 2018	[20]
Trametinib	Targets an allosteric pocket adjacent to the ATP-binding site of MEK1 and MEK2	FDA Approved, 2013	Approved with Dabrafenib in 2022	[16,51]
Cobimetinib	Targets an allosteric pocket adjacent to the ATP-binding site of MEK1 and MEK2	FDA Approved, 2015	Approved in combination with Vemurafenib in 2015	[16]
Binimetinib	Reversibly inhibits MEK1 and MEK2	FDA Approved, 2018	Approved in combination with Encorafenib in 2018	[20]
Defactinib	Inhibits the phosphorylation of FAK	Clinical trial phase ii for uveal melanoma (with combination of RAF/MEK inhibitor VS-6766)		[47,48]
Sorafenib	Pan-RAF inhibitor targeting CRAF and BRAF	Success in pre-clinical mouse models and phase i study with selumetinib on hepatocellular carcinoma patients		[50]
AZ628	Pan-RAF inhibitor targeting CRAF, BRAF, and BRAFV600E	Success in pre-clinical mouse models		[50]
Selumetinib	Non-ATP competitive MEK1 and MEK 2 inhibitor	Success in clinical trials	Phase I trial with sorafenib on hepatocellular carcinoma patients shows promising effects	[50]
Tovorafenib	CNS-penetrant, type II pan-RAF inhibitor	Successful safety profile in phase I trial for patients with melanoma		[51]
Bemcentinib	Blocks AXL autophosphorylation and induce apoptosis	Phase 1b/2 clinical trial comparing its efficacy with pembrolizumab or dabrafenib/trametinib alone on stage III or IV unresectable melanoma	Also known as BGB324 or R428	[61,62]
Crizotinib	ATP competitive inhibitor of Met and ALK kinases	Shown to be effective with afatinib on cutaneous melanoma patient cell models	Has FDA approval for NSCLC, and combination studies with crizotinib on lung cancer and mesothelioma showed strong efficacy	[70,71]
Tivantinib	Non-ATP competitor that inhibits MET selectively	A phase I trial with sorafenib on melanoma and other solid tumors showed promising results		[70]
Quercetin	STAT3 inhibitor that inhibits MET activation through FAS inhibition	Shows promising success in pre-clinical melanoma models		[70]
Afatinib	Irreversibly inhibits ERBB family receptors including ERBB3	Shown to be effective with crizotinib on cutaneous melanoma patient cell models	Combination studies with crizotinib on lung cancer and mesothelioma showed strong efficacy	[71]
PHA665752	Blocks MET phosphorylation	Showed success with pre-clinical studies from melanoma patient tumor samples		[72]
11a-1	Specifically inhibits SHP2, blocking ERK1/2 and AKT activation	Showed success with pre-clinical tests from melanoma cell lines		[36]
SCH772984	Potent ATP-competitive compound that inhibits ERK1 and ERK2	Successfully blocked proliferation in melanoma models, including those with BRAFi/MEKi resistance		[37]
Hydroxychloroquine	Inhibitor of autophagy by impairing lysosomal function	Phase 1 trial testing hydroxycholorquine and vemurafenib in melanoma is completed		[40]
Palbociclib	Highly selective ATP-competitive inhibitor of CDK4 and CDK6	Preclinical trial combination with irradiation on donor skin cancer cells showed cell cycle arrest	FDA approved for breast cancer	[56,57]
Pembrolizumab	Monoclonal antibody that blocks programed death-ligand 1 (PD-1) on T-cell surfaces	FDA approval for metastatic melanoma in 2014 and stage iib/c melanoma in 2021		[87]
Ipilimumab	Human monoclonal antibody against CTLA-4	Approved by FDA for unresectable, metastatic melanoma in 2011 and in combination with nivolumab in 2015	Many clinical trials in combination with other drugs are in progress	[19]
Tremelimumab	Human monoclonal antibody against CTLA-4	Multiple phase I combination clinical trials showed effectiveness in advanced melanoma		[19,89]
BCD-145	Human monoclonal antibody against CTLA-4	Multiple phase i clinical trials of solo or combination BCD-145 treatments on advanced melanoma are undergoing		[19]
Nivolumab	Monoclonal antibody that blocks programed death-ligand 1 (PD-1) on T-cell surfaces	Approved solo for metastatic melanoma in 2014 and in combination with ipilimumab in 2015	Shows success with relatimab in a phase ii/iii clinical trial	[19,87]
Avelumab	Human monoclonal antibody against PD-L1	A phase I trial showed promising results in melanoma	Approved by FDA to treat Merkel cell carcinoma	[19]
Drurvalumab	Human monoclonal antibody against PD-L1	Multiple phase I combination clinical trials showed effectiveness in advanced melanoma		[19,89]
Cemiplimab	Human monoclonal antibody against PD-1	Multiple clinical trials retesting the efficacy of cemiplimab in melanoma	Approved by FDA in 2018 for cutaneous squamous cell carcinoma	[19]
Atezolizumab	Human monoclonal antibody against PD-1	Approved in combination with cobimetinib and vemurafenib for advanced melanoma in 2022		[19]
Cosibelimab	Human monoclonal antibody against PD-L1	Phase iii trials in cutaneous squamous cell carcinoma are being investigated for their efficacy		[19]
Tebentafusp	A bispecific gp100 T-cell engager	Multiple phase I combination clinical trials showed effectiveness in advanced melanoma		[89]
Relatlimab	Anti-LAG-3 antibody	A phase ii/iii trial in combination with nivolumab on advanced melanoma shows promising results		[87,93]
RO7247669	Anti-PD-1 and LAG-3 bispecific antibody	A phase I clinical trial is evaluating its efficacy in solid tumors such as melanoma		[95,96]
Lifileucel	Autologous TIL therapy product	FDA approved in 2024 to treat patients with unresectable or advanced melanoma	Also known as Amtagvi	[109]
Canvaxin	Allogenic whole-cell melanoma vaccine made of three cell lines	Multiple phase iii trials failed to show benefit over placebo		[142]
Melacine	Allogenic whole-cell melanoma vaccine made of two cell lines	Multiple phase iii trials failed to show significant benefit		[139]
Oncept	Xenogenic DNA caxxine targeting tyrosinase	USDA approved for stage ii/iii canine oral melanoma but has limited efficacy		[147]
FixVac	Encodes RNA targeting 4 TAAs: NY-ESO, MAGE-A3, tyrosinase, and TPTE	A phase I trial showed promising results in advanced melanoma patients		[149]
mRNA-4157	Encodes 34 neoantigens	A phase 2b for resected melanoma		[152]
Aldesleukin (IL-2)	Stimulates immune cells with IL-2 receptors	FDA approved for melanoma in 1998, commonly used with ACT to improve response rates		[112]
Talimogene Laherparpvec	Oncolytic viral therapy that selectively replicates in tumor cells, injecting with GM-CSF	Approved by FDA in 2015 for local treatment of unresectable stage iii/iv melanoma		[180]
Dacarbazine	Chemotherapy drug that targets cancer cell’s DNA	Approved by FDA for melanoma in 1975		[22]

## Abbreviations

AI: artificial intelligence; AJCC: American Joint Committee on Cancer; ACT: adoptive cellular therapy; BCG: bacillus Calmette–Guerin; BRAF: Raf proto-oncogene serine-threonine protein; CAR: chimeric antigen receptor; CLTA-4: cytotoxic T lymphocyte-associated protein 4; CSPG4: chondroitin sulphate proteoglycan 4; DC: dendritic cell; DETOX: detoxified Freund’s adjuvant; GM-CSF: granulocyte–macrophage colony-stimulating factor; ICI: immune checkpoint inhibitor; LAG-3 (CD223): Lymphocyte activation gene-3; MHC: major histocompatibility complex; MAPK: mitogen-activated protein kinase; ML: machine learning; NF1: neurofibromin-1 mutant; NRAS: NRAS proto-oncogene GTPase; OS: overall survival; PD-1: programmed cell death protein; PD-L1: programmed cell death-ligand 1; PFS: progression-free survival; scFv: single-chain variable fragment; RFS: relapse-free survival; TAAs: tumor-associated antigens; T_EX_: exhausted T cells; T_REGS_: regulatory T cells; TCRs: T-cell receptors; TEAs: treatment-emergent adverse events; TILs: tumor-infiltrating lymphocytes; T-VEC: talimogene laherparepvec; TME: tumor microenvironment; UV: ultraviolet; V_H_: antibody-variable heavy chain; V_L_: antibody-variable light chain; WT: wildtype.
